# Sodium-Glucose Co-transporter 2 Inhibitor-Associated Euglycemic Diabetic Ketoacidosis in a Recipient of Concurrent Pancreas-Kidney Transplantation With Type 1 Diabetes Mellitus

**DOI:** 10.7759/cureus.89866

**Published:** 2025-08-12

**Authors:** Takahiro Kawaji, Tsukasa Jinno, Satoshi Komatsu, Naohide Kuriyama, Tomoyuki Nakamura

**Affiliations:** 1 Department of Anesthesiology and Critical Care Medicine, Fujita Health University School of Medicine, Toyoake, JPN

**Keywords:** euglycemic diabetic ketoacidosis, ketone level, organ transplantation, sodium-glucose co-transporter 2 inhibitors (sglt2is), type 1 diabetes (t1d)

## Abstract

Currently, sodium-glucose co-transporter 2 inhibitors (SGLT2is) are used frequently in a range of patients, including those with type 1 diabetes mellitus, raising concerns regarding the potential increased risk of SGLT2i-associated euglycemic diabetic ketoacidosis (EDKA). We report the case of a patient with type 1 diabetes who developed SGLT2i-associated EDKA during the perioperative period of concurrent pancreas and kidney transplantation procedure. A 41-year-old woman taking an SGLT2i underwent concurrent pancreas and kidney transplantation from a brain-dead donor. Acidosis was noted during surgery, which improved temporarily after the pancreas was transplanted. The pancreatic allograft was resected because of twisting of the anastomotic vessels, and acidosis recurred. The patient’s blood glucose levels were consistently within the normal range. Blood ketone monitoring was initiated, and the patient was diagnosed with EDKA. Patients with type 1 diabetes taking SGLT2is who undergo emergency surgery are at risk of EDKA and should continually monitor their ketone levels.

## Introduction

Sodium-glucose co-transporter 2 inhibitors (SGLT2is) are antidiabetic drugs that promote urinary glucose excretion by inhibiting SGLT2 in the proximal renal tubules [1] and have recently been recognized for their benefits in slowing the progression of heart failure and chronic kidney disease [2,3]. Euglycemic diabetic ketoacidosis (EDKA) is an adverse event associated with SGLT2is, characterized by near-normal blood glucose levels (< 250 mg/dL), severe metabolic acidosis (pH < 7.3, blood bicarbonate < 18 mEq/L), and ketonemia [4]. SGLT2i-associated EDKA is triggered by surgical stress and fasting, and its incidence in perioperative patients has been increasingly reported in recent years [5], with estimated rates of 0.17% for nonemergent procedures and 1.1% for emergent procedures [6].

In patients with type 1 diabetes, endogenous insulin production is virtually absent, as indicated by undetectable C-peptide levels, rendering them entirely dependent on exogenous insulin to prevent ketoacidosis. Although SGLT2is are typically used in patients with type 2 diabetes and are not approved for those with type 1 diabetes in many countries, they have been used off-label as adjunctive therapy to insulin in some cases, raising concerns regarding an increased risk of SGLT2i-associated EDKA [7]. Herein, we report the case of a patient with type 1 diabetes who developed SGLT2i-associated EDKA during the perioperative period of a concurrent pancreas and kidney transplantation procedure.

## Case presentation

Preoperative course

The patient was a 41-year-old woman (height, 156 cm; weight, 49 kg) who had been diagnosed with type 1 diabetes at the age of 9 years and had been receiving insulin therapy long-term. A few years prior, owing to worsening glycemic control, she was switched to insulin pump therapy via continuous subcutaneous insulin infusion (CSII). Additionally, ipragliflozin, a sodium-glucose cotransporter 2 inhibitor (SGLT2i), was introduced to improve glycemic control, particularly to reduce hemoglobin A1c (HbA1c) levels. With this combination therapy, her HbA1c was well maintained at 5.5% (reference range: 4.6-6.2%). More recently, her estimated glomerular filtration rate had declined to below 30 mL/min/1.73 m² (from > 60 mL/min/1.73 m²).

She was urgently admitted to our hospital after a brain-dead donor became available for concurrent pancreas and kidney transplantation. She had taken her last dose of ipragliflozin approximately 30 h prior to the start of surgery. Upon admission, the patient was placed on preoperative fasting, during which her basal insulin dose of 17 units was maintained via CSII. CSII was discontinued immediately prior to the operation, which was scheduled for the following day.

Concurrent pancreas-kidney transplantation

The patient underwent intraoperative electrocardiography, peripheral oxygen saturation measurement, and invasive arterial pressure monitoring. General anesthesia was induced using a rapid induction and intubation technique with target-controlled infusions of propofol (3 μg/mL), fentanyl (100 μg), remifentanil (0.2 μg/kg/min), and rocuronium (40 mg). Anesthesia was maintained with continued target-controlled infusion of propofol, remifentanil (0.2 μg/kg/min), and intermittent administration of rocuronium. A constant-rate infusion of a 2.5% glucose solution was initiated at the start of surgery. Exogenous insulin was withheld intraoperatively to assess pancreatic graft function. The patient’s blood glucose levels were monitored throughout the procedure and ranged between 100 and 200 mg/dL. Her hemodynamics were stabilized with 0.03 μg/kg/min of noradrenaline, but persistent high anion gap metabolic acidosis was observed during the operation. Arterial blood gas analysis results were: pH, 7.22 (7.35-7.45); HCO3−, 15.4 (22.0-26.0) mmol/L; base excess, -11.4 (-2.0-2.0) mmol/L; anion gap, 16.6 (10.0-15.0) mEq/L (Table 1). A kidney graft was transplanted into the left iliac fossa and a pancreatic graft into the right iliac fossa. Ultrasound examination of the surgical field showed good blood flow in both grafts. The durations of the surgery and anesthesia were 363 and 447 min, respectively. Blood loss and urine output were 203 and 250 mL, respectively. The patient was administered 2,900 mL of crystalloid and 750 mL of 5% albumin.

**Table 1:** Blood gas analysis during the transplantation surgery, after the patient’s transfer to the ICU and post-pancreatectomy

Parameter	Reference range	During transplant surgery	ICU transfer	Post-pancreatectomy
pH	7.35-7.45	7.22	7.41	7.29
pCO_2 _(mmHg)	35.0-45.0	37.7	30.3	36.2
HCO_3_^-^ (mmol/L)	22.0-26.0	15.4	19.3	17.6
Base excess (mmol/L)	-2.0 to 2.0	-11.4	-5.5	-9.2
Sodium (mmol/L)	135.0-145.0	138.0	142.3	144.3
Potassium (mmol/L)	3.5-5.0	4.3	3.9	4.1
Chloride (mmol/L)	98.0-108.0	106.0	108.4	108.2
Anion gap (mEq/L)	10.0-15.0	16.6	14.6	18.5
Lactate (mg/dL)	4-16	13	4	5
Glucose (mg/dL)	70-110	114	160	184

Postoperative course

The patient was transferred to the intensive care unit (ICU) while remaining intubated and sedated with propofol at 4 mg/kg/h. Blood glucose levels were measured hourly using an arterial blood gas analyzer to detect any pancreatic graft dysfunction. The metabolic acidosis observed during surgery had temporarily improved at the time of ICU admission (Table 1), but worsened 5 h later, with the following findings: pH: 7.28 (7.35-7.45); HCO3−: 15.4 (22.0-26.0) mmol/L; base excess: -10.0 (-2.0-2.0) mmol/L; anion gap: 17.2 (10.0-15.0) mEq/L. Bedside ultrasonography in the ICU confirmed an adequate intravascular volume status. High blood glucose fluctuations, which typically indicate pancreatic graft dysfunction, were not observed. Doppler ultrasonography performed 10 h after ICU admission detected venous graft thrombosis in the transplanted pancreas, and surgical re-examination was promptly performed. Twisting of the anastomotic vessels was found to be the cause of the vascular thrombosis, and allograft pancreatectomy was performed.

**Figure 1:**
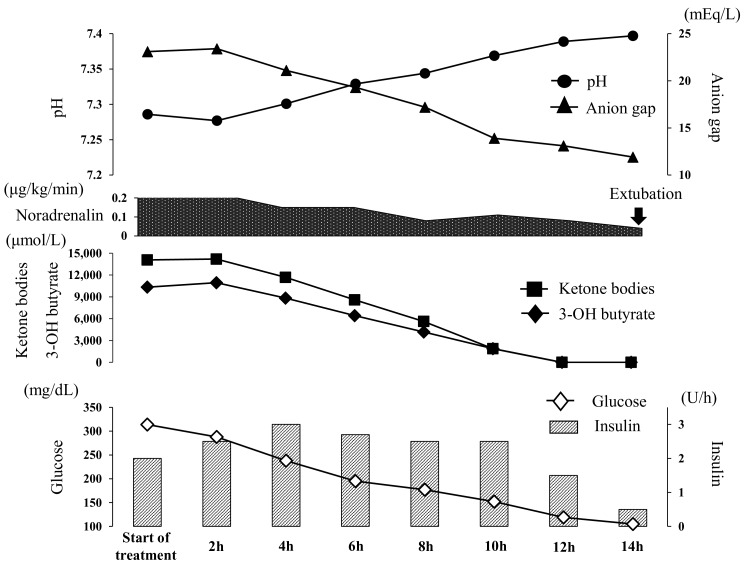
Treatment course for ketoacidosis in the ICU The patient was started on treatment with insulin drips, leading to closure of the anion gap, acidosis, a rapid decline in ketone bodies, and hemodynamic stability. Twelve hours after treatment initiation, the patient’s metabolic acidosis had resolved, and she was extubated. 3-OH butyrate: 3-hydroxybutyrate

After the surgery, the patient’s metabolic acidosis worsened (Table 1), and her hemodynamic status became unstable. Urine ketones, assessed using the nitroprusside method, were markedly positive. Blood ketone body levels were also extremely elevated at 14,077 (<130 μmol/L), including 3-hydroxybutyrate at 10,338 (< 85 μmol/L) and acetoacetate at 3,739 (< 55 μmol/L). The patient was diagnosed with EDKA, presumably related to her SGLT2i therapy. She was started on an insulin pump and infused with a 10% glucose solution. By 12 h after the initiation of treatment, the anion gap had closed and her metabolic acidosis had resolved (Figure 1). The patient was weaned off the ventilator on postoperative day 2. Her blood ketone body level decreased to 342 μmol/L, and she was discharged from the ICU on postoperative day 3.

## Discussion

Mechanism of EDKA with SGLT2is

The general pathophysiology of diabetic ketoacidosis (DKA) involves severe insulin deficiency and an increase in insulin-antagonist hormones such as glucagon, leading to hyperglycemia because of impaired glucose utilization and increased ketone body production from excess lipolysis [7]. The pathophysiology of SGLT2i-associated ketoacidosis is complex but likely involves inhibition of SGLT2 in the proximal renal tubules, which promotes glucosuria and thereby reduces plasma glucose levels [5]. SGLT2i also independently stimulates α-cell activation and increases plasma glucagon concentrations [8], leading to increased ketogenesis in the liver. In patients with type 1 diabetes, adjunctive therapy with SGLT2is may reduce total insulin doses and increase ketoacidosis events because of elevated ketone levels when low doses of insulin are insufficient to inhibit lipolysis in surrounding adipose tissues [9]. 

Our case differed from the usual clinical course because SGLT2i-associated EDKA developed during the perioperative period of concurrent pancreas and kidney transplantation. High-anion-gap metabolic acidosis was observed during the operation; therefore, it was assumed that the patient’s ketoacidosis had progressed. The triggering factors for EDKA in this case included type 1 diabetes, reduced carbohydrate intake, SGLT2i use, and insulin pump therapy [9]. Although insulin pump therapy was combined with an SGLT2i to improve glycemic control, this combination may have further increased the risk of ketoacidosis. Moreover, the inappropriate discontinuation of exogenous insulin during the transplant procedure likely led to relative insulin deficiency and worsening acidosis.

Diagnostic considerations

Following pancreatic transplantation, the patient’s acidosis temporarily improved after she was admitted to the ICU. This was likely because her EDKA was masked by a release of insulin from the pancreatic graft. Allograft pancreatectomy was later performed to treat twisting of the anastomotic vessels, resulting in depletion of insulin influx and recurrence of ketoacidosis. We began monitoring the patient’s ketone bodies at this time and ultimately identified EDKA as the cause of her high-anion-gap metabolic acidosis. However, polyuria, a clinical sign associated with EDKA, was not observed in this case. The exact cause, therefore, remains unknown; however, it may have been related to preoperative fasting, insensible perspiration during the surgery, intravascular dehydration caused by fluid loss during the surgery, or drug reactivity to the grafted kidney. 

To the best of our knowledge, there have been no reports in the literature of patients with type 1 diabetes who developed EDKA during the perioperative period of pancreatic transplantation. SGLT2i-associated EDKA may not follow its typical course during this period, making diagnosis potentially challenging. Ketone monitoring and repeated blood gas analyses are key to detecting perioperative DKA. Urine ketone (i.e., nitroprusside) testing often underestimates the severity of ketoacidosis because it does not detect the presence of 3-hydroxybutyrate, the main ketone body produced during ketoacidosis. Whenever possible, point-of-care blood testing devices that can rapidly measure 3-hydroxybutyrate should be used [10,11].

Timing of SGLT2i discontinuation and perioperative management recommendations

Because the effects of SGLT2is are long-lasting, it has been recommended that these drugs be discontinued at least 24 hours before elective surgeries [12]. However, since there have been reports of EDKA occurring even when these drugs were suspended the day before surgery, a new recommendation for a 72-hour drug suspension period has been proposed, and it is currently under evaluation [13,14]. Transplantation surgery from brain-dead donors typically requires emergency hospitalization of the recipient on the day before, or even the day of, the procedure, which may make it difficult to ensure adequate drug discontinuation time. Anesthesiologists, who play a central role in perioperative management, should be aware of the potential for EDKA and monitor ketone body levels. Intravenous insulin and glucose should be administered to maintain appropriate blood glucose levels and prevent ketosis. When feasible, preoperative blood ketone screening and the implementation of a perioperative protocol for SGLT2i cessation may help reduce the risk of EDKA.

Monitoring exocrine pancreatic function can serve as an early indicator of pancreatic rejection. Blood glucose levels can also be measured easily and frequently early after transplantation. Graft thrombosis or rejection should be suspected if sudden increases in blood glucose levels are observed during the procedure. However, caution is required when administering SGLT2is, as these can cause blood glucose fluctuations. 

In this case, twisting of the anastomotic vessels led to a thrombus in the pancreatic graft, resulting in pancreatic graft necrosis during the procedure. Although insulin secretion likely decreased abruptly, the glycosuric effect of the SGLT2i may have persisted, potentially affecting blood glucose fluctuations. As a result, there was no sudden rise in the patient’s blood glucose levels, making this difficult to detect. Therefore, in patients taking SGLT2is during the perioperative period of pancreatic transplantation, diagnosis should be attempted using other indicators such as increases in amylase and lipase levels, or imaging diagnostics such as ultrasound and contrast-enhanced ultrasound.

## Conclusions

Importantly, any fasting patient with type 1 diabetes, as diagnosed by a low C-peptide level, should be treated with intravenous insulin and glucose during the perioperative period to reduce the risk of ketosis. SGLT2is should be discontinued perioperatively, and insulin should be used instead to prevent ketoacidosis. For patients with type 1 diabetes taking SGLT2is who undergo emergency surgery, it is important to be aware of the risk of EDKA and to carefully monitor ketone levels. Fluctuations in blood glucose levels, which are typically used to monitor pancreatic graft function, may be affected by SGLT2is. Anesthesiologists and transplant physicians should maintain a high index of suspicion for EDKA, even in the absence of hyperglycemia or markers of graft dysfunction.
